# Synchronous Acute Ischemic Stroke and Acute Myocardial Infarction: A Case Report

**DOI:** 10.7759/cureus.80054

**Published:** 2025-03-04

**Authors:** Ivan Grela, Thomas R Peterson

**Affiliations:** 1 Emergency Medicine, Florida Atlantic University Charles E. Schmidt College of Medicine, Boca Raton, USA; 2 Emergency Medicine, St. Mary's Medical Center, West Palm Beach, USA

**Keywords:** acute st-elevation myocardial infarction, ais (acute ischemic stroke), cardiocerebral infarction, emergency and acute care, stroke management

## Abstract

The synchronous presentation of acute ischemic stroke (AIS) and acute myocardial infarction (AMI), termed concurrent cardiocerebral infarction (CCI), is a rare but fatal condition that poses challenges in diagnosis and management. This case report contributes to the limited literature on the successful management of a patient presenting with CCI while emphasizing the risk-benefit balance in thrombolysis and dual antiplatelet therapy. We report a 51-year-old male who presented to the emergency department with a stroke alert after experiencing right-sided flaccid paralysis and aphasia. National Institute of Health Stroke Scale (NIHSS) score on arrival was 24. An initial CT angiogram of the head confirmed the presence of an occluded left internal carotid artery (ICA) terminus with no flow in the left middle cerebral artery (MCA). Tenecteplase 0.25mg/kg was administered, and the patient subsequently underwent thrombectomy. An EKG after undergoing thrombectomy revealed ST elevations in the anteroseptal leads V1-V4. After the thrombectomy, the patient was transferred to a facility with interventional cardiology, where he underwent percutaneous coronary aspiration thrombectomy with drug-eluting stent placement into the proximal and mid-left anterior descending artery. The patient received 300mg rectal aspirin and IV cangrelor. He was continued on aspirin/clopidogrel dual therapy, and the patient was subsequently discharged and instructed to follow up with a cardiologist. This case report underscores the necessity of a holistic approach to management guidelines for patients presenting with synchronous AIS and AMI due to the potential complications associated with the treatment of either AIS or AMI.

## Introduction

Acute ischemic stroke (AIS) and acute myocardial infarction (AMI) occurring synchronously is a relatively rare presentation. A recent study from 2022 conducted with data from a hospital registry in Singapore found that between 2014 and 2018, the incidence of a concurrent cardiocerebral infarction (CCI) was 0.29% [1]. Another study published in 2021 with data from a hospital registry in the Philippines found that the prevalence of a synchronous CCI was 0.25% [2]. There is no literature reporting the prevalence of CCI in the United States. 

CCI can be categorized into two subtypes, depending on the timing of the occurrence. Synchronous CCI occurs when the patient presents with simultaneous AMI and AIS, whereas metachronous CCI occurs when one event precedes the other within 72 hours, regardless of the order of presentation of AMI and AIS [3]. In the Philippines, synchronous CCI has a prevalence rate of 0.9%, whereas metachronous CCI has a prevalence rate of 0.9% to 12.7% [3].

Some of the suggested etiologies of CCI include embolic and hypotensive causes [1]. Some of the mechanisms cited as embolic causes include a hypokinetic myocardial segment, thrombosis in the presence of a right-to-left shunt, atrial fibrillation, and a ventricular thrombus, while mechanisms under hypotensive causes include atherosclerotic stenosis, aortic dissection, myocardial infarction, and insular infarct with arrhythmia [1]. The insular cortex is a brain region involved in regulating sympathetic and parasympathetic signaling, and studies show that arrhythmias are more frequent when cerebral ischemia involves the insular cortex than when the insular cortex is not involved [4]. 

While the management of either AIS or AMI is well established, there are no clear guidelines for the management of a patient presenting with synchronous AIS and AMI. In addition, aspirin and clopidogrel, used in the management of AMI, can lead to hemorrhagic conversion of an AIS, complicating management further [5]. Further complicating the management of patients presenting with CCI is the complications that can develop when treating each condition separately. For example, if treatment to salvage the myocardium is initiated first, the delayed management of the ischemic stroke will result in worsening disability, whereas if treatment to salvage the brain tissue is initiated first, the delayed management of the myocardial infarction can result in hypotension or arrhythmias [6]. 

This case report of a 51-year-old man presenting with a concurrent CCI underscores the need for guidelines on the management of patients presenting with CCIs.

## Case presentation

A 51-year-old male with a past medical history of type 2 diabetes mellitus, hypertension, and hyperlipidemia, non-adherent with all of his home medications, presented to the emergency department as a stroke alert after experiencing right-sided flaccid paralysis, left gaze deviation, and aphasia. Symptoms began one hour prior to arrival. Further history could not be obtained due to the patient's aphasia. The patient's wife provided additional history, stating the patient's brother had a triple bypass at the age of 55, the patient's father died at the age of 52 from a myocardial infarction, and the patient's mother also suffered a myocardial infarction. 

On examination, the patient's vitals were: heart rate 94 beats/minute, blood pressure 132/87 mmHg, SpO2 98% on room air, respiratory rate 20 breaths/minute. Cardiovascular, respiratory, and abdominal examinations were unremarkable. Neurological examination revealed global aphasia, right upper and lower extremity flaccid paralysis, right lower facial droop, and left-sided gaze deviation. The National Institute of Health Stroke Scale (NIHSS) on arrival was 24. Point-of-care glucose was 336 mg/dL on arrival. 

**Figure 1:**
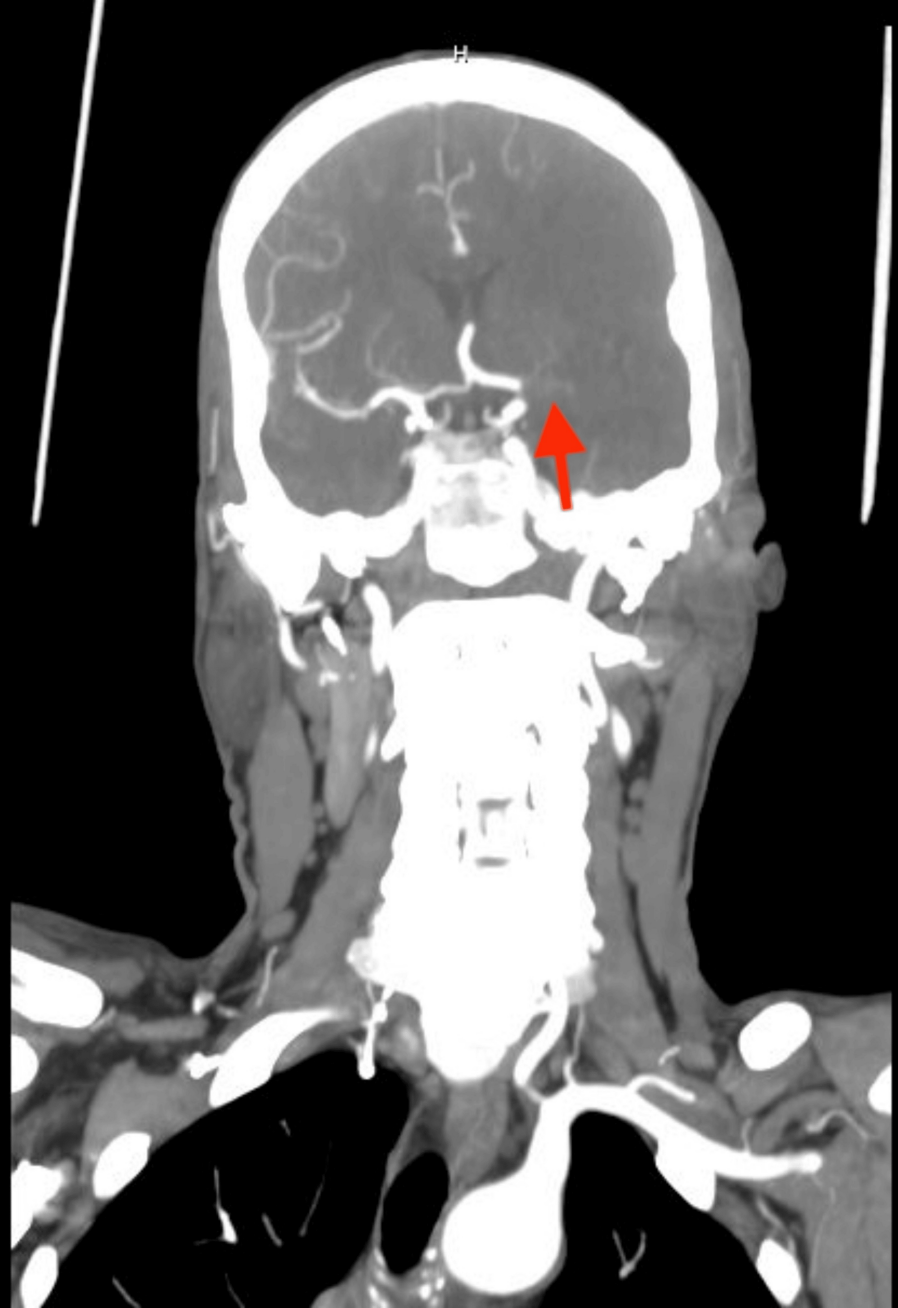
CT head angiogram shows no flow in the left middle cerebral artery (red arrow)

**Figure 2:**
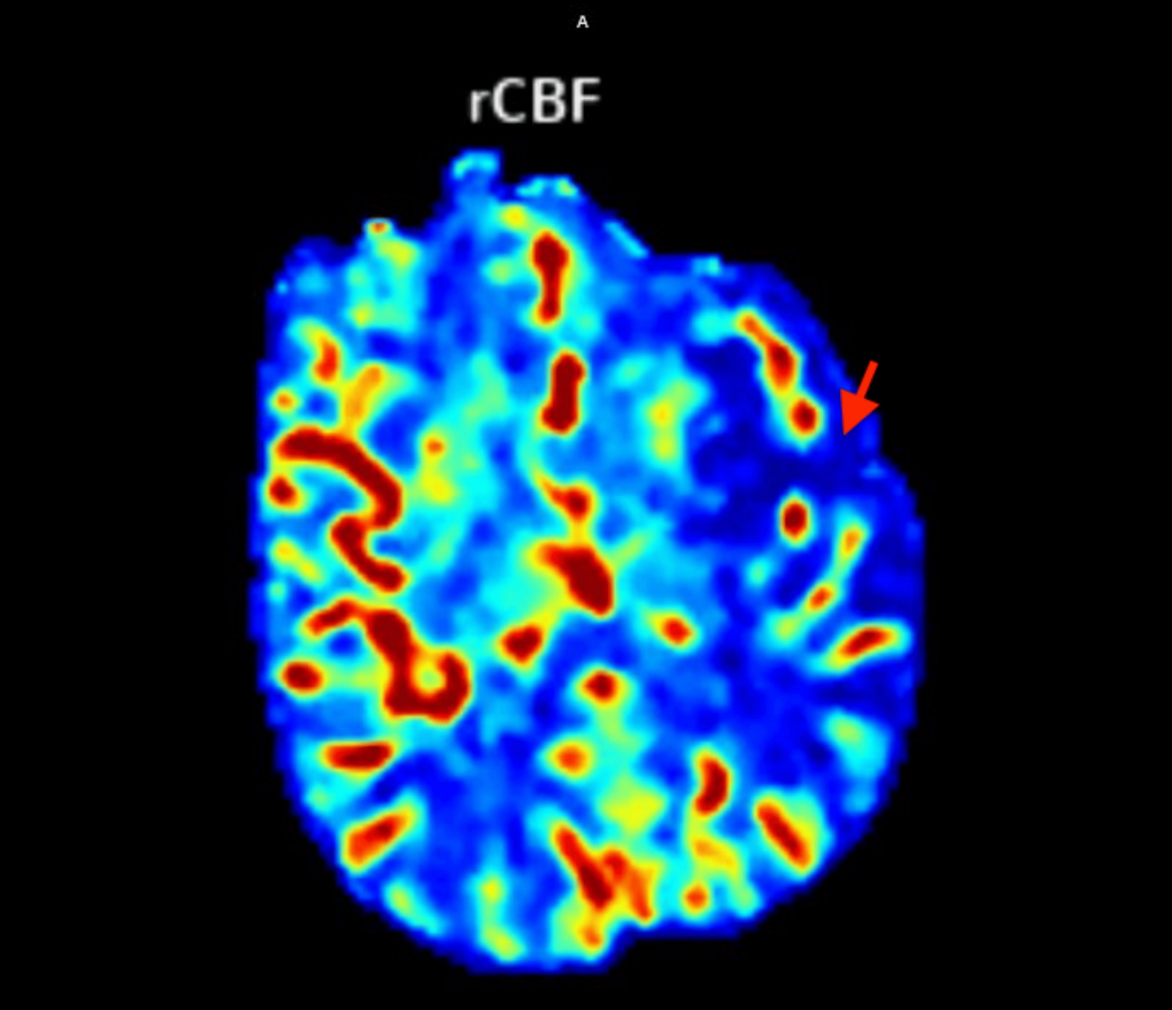
CT cerebral perfusion scan showing a large area of ischemia throughout the middle cerebral artery distribution (red arrow) rCBF - regional cerebral blood flow

Complete blood count, basic metabolic panel, and prothrombin time/ partial thromboplastin time (PT/PTT) studies were unremarkable. An initial CT scan of the head without contrast showed a slight asymmetric hyper-density in the left internal carotid artery (ICA) terminus, suggestive of an intraluminal thrombus. CT angiograms of the head and neck with contrast (Figure 1) and CT cerebral perfusion scan with contrast (Figure 2) revealed an occluded left ICA terminus with no flow in the left middle cerebral artery (MCA) and a large area of ischemia throughout the MCA distribution. Tenecteplase 0.25mg/kg was administered while the patient was in CT. The patient was subsequently sent for aspiration thrombectomy. A consult was placed for the neurology team and the critical care team.

**Figure 3:**
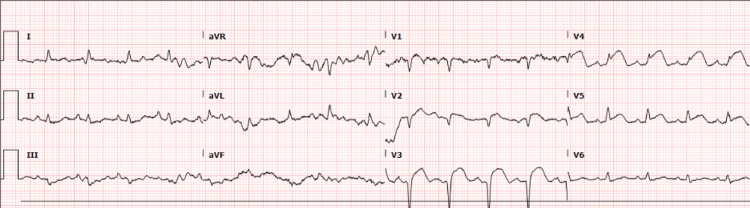
Initial EKG demonstrating ST elevations in leads V3, V4

After the patient underwent aspiration thrombectomy, an EKG was performed, which demonstrated ST elevations in leads V3 and V4, consistent with an anteroseptal infarct (Figure 3). The EKG had not been performed on arrival to the hospital since the patient arrived with a stroke alert and, in accordance with the hospital protocol, the patient was sent for imaging and subsequent thrombectomy. 

**Figure 4:**
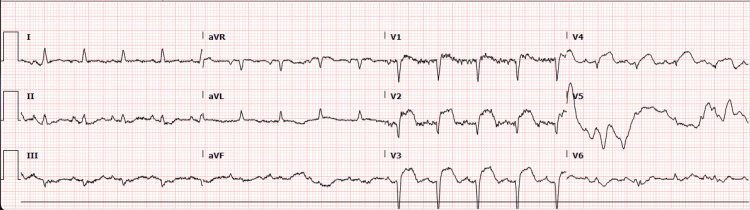
EKG with ST elevations in the anteroseptal leads V1-V4

Once the patient arrived at the intensive care unit, insulin was started to manage the elevated glucose. A repeat EKG revealed ST elevations in the anteroseptal leads V1-V4 (Figure 4). An echocardiogram was performed, which showed a normal bubble study and an estimated left ventricular ejection fraction of 30-35% with severe anterior/anteroseptal/apical hypokinesis. The cardiology team was consulted, and recommended that the patient be transferred to a facility with interventional cardiology. The interventional cardiologist, in consultation with the neurologist, determined percutaneous coronary aspiration thrombectomy was needed due to the low left ventricular ejection fraction and severe hypokinesis of the myocardium despite the high risk associated with the procedure. The patient's family was agreeable to the plan. The patient underwent successful percutaneous coronary aspiration thrombectomy with drug-eluting stent placement to the proximal and mid-left anterior descending artery. The patient received 300mg rectal aspirin and intravenous cangrelor, as indicated for the percutaneous coronary intervention. After percutaneous coronary intervention, the patient was transferred to the cardiovascular intensive care unit, where he was continued on a dual therapy consisting of aspirin 81mg oral daily and clopidogrel 75mg oral daily. 

**Figure 5:**
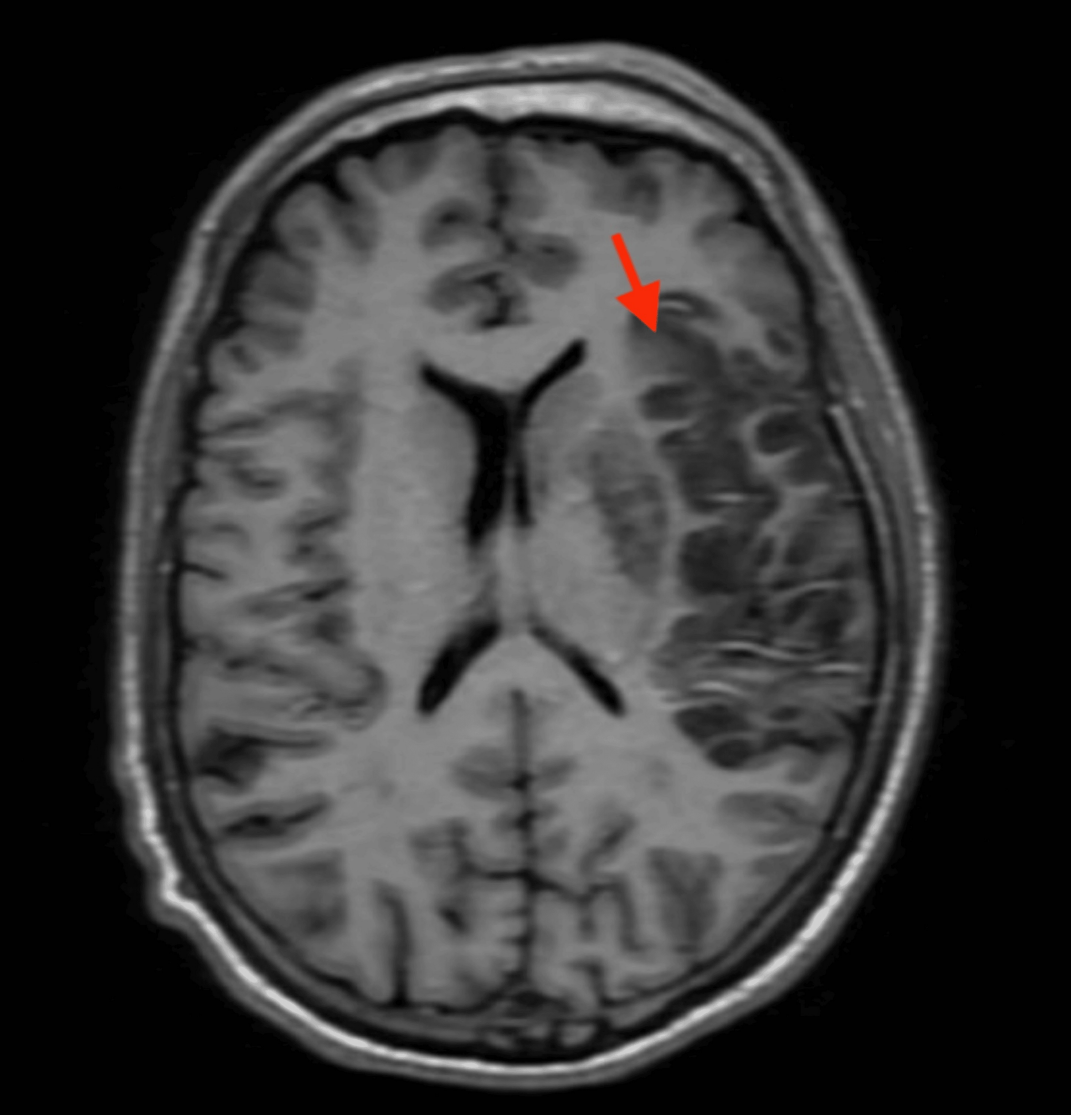
T1 FLAIR MRI of head shows area of hypo-intensity in the left middle cerebral artery distribution of the brain (red arrow) FLAIR - fluid attenuated inversion recovery

MRI of the brain within 24 hours of the stroke revealed acute ischemia in the left middle cerebral artery (MCA) distribution (Figure 5) with no evidence of hemorrhagic conversion. The patient was eventually transitioned to clopidogrel 75mg oral daily and apixaban 5mg oral twice a day therapy due to the high embolic risk secondary to the early-onset cardiovascular disease and family history of early-onset cardiovascular disease. The patient improved speech throughout the hospital stay and regained movement in the right upper and lower extremities, and he was eventually discharged home to follow up with a cardiologist. No follow up NIHSS score was provided in the documentation.

## Discussion

There is currently a lack of literature detailing the management of a patient presenting with a CCI. Since both AIS and AMI are time-sensitive conditions, delayed interventions may result in permanent disability or even death. Some of the dilemmas encountered in patients who present with CCI include: 1) different dosages of fibrinolytic therapy needed to treat AIS and AMI [7], 2) use of PCI requires heparin and dual-antiplatelet therapy, both which increase the risk of hemorrhagic conversion in AIS [8], and 3) risk of myocardial rupture when using thrombolytics in a patient with an acute STEMI [9]. A review of 25 cases of patients presenting with CCI showed that seven (28%) used mechanical thrombectomy, 15 (60%) underwent PCI, and nine (36%) received alteplase, showing that the management for CCI is individualized [7]. 

A suggested approach to the management of CCI is treatment of both vascular territories with IV-tPA at 0.9mg/kg (maximum of 90mg) infused over one hour, with 10% of the total dose administered as an initial IV bolus for one minute, followed by PCI if indicated and later assessing the need for mechanical thrombectomy [10]. A similar algorithm has been proposed in a different study, favoring fibrinolysis as an initial treatment strategy in hemodynamically stable patients, followed by endovascular procedures [11]. 

The delay in detection of AMI in the patient in this case report was due to the global aphasia associated with his AIS, further complicating the management. One study on patients with CCI found that 83% of patients presented with focal neurological deficits, while 17% presented with AMI symptoms, including dyspnea and chest pain [2]. To avoid delaying detection of a possible concurrent AMI in patients presenting with AIS, routine EKGs should be performed on arrival to the hospital for all patients who present with a stroke alert.

## Conclusions

Cardiocerebral infarction (CCI) is a relatively rare but deadly condition that necessitates the establishment of clear management guidelines due to its high mortality and challenges in treatment strategies, including the risk of hemorrhagic conversion. This case report highlights the challenges in identifying and managing a patient presenting with CCI. When encountering a patient with a CCI, it is important to determine the hemodynamic stability of the patient, the time since onset of symptoms, and possible etiologies causing the presentation to optimize the management of the patient. In this case, the AIS was managed first per hospital protocol since the patient arrived with a stroke alert, and the AMI was subsequently managed. This case report contributes to the limited existing literature of patients presenting with CCIs, highlighting a case where a patient presenting with CCI was successfully managed while underscoring the need for guidelines managing of patients with CCIs. Further research needs to be conducted to determine optimal fibrinolytic dosage, determining whether to salvage the brain tissue or myocardium first, and subsequent anticoagulation strategies. 
